# Prioritizing emergency department antibiotic stewardship interventions for skin and soft tissue infections using judgment analysis

**DOI:** 10.1017/ice.2024.211

**Published:** 2025-03

**Authors:** Meggie Griffin, Kimberly C. Claeys, Rebecca J. Schwei, Roger L. Brown, Michael S. Pulia

**Affiliations:** 1 BerbeeWalsh Department of Emergency Medicine, University of Wisconsin-Madison School of Medicine and Public Health, Madison WI, USA; 2 Department of Practice, Sciences, and Health Outcomes Research, University of Maryland School of Pharmacy, Baltimore MD, USA; 3 University of Wisconsin-Madison School of Nursing, Madison WI, USA

## Abstract

**Objective:**

Skin and soft tissue infections (SSTIs) account for over 2.8 million annual emergency department (ED) visits and often result in suboptimal antibiotic therapy. The objective of this study was to evaluate a set of interventions in minimizing inappropriate prescription of antibiotics for presumed SSTIs in the ED.

**Design:**

Case vignette survey.

**Participants:**

A national sample of emergency medicine (EM) physicians.

**Methods:**

Each vignette described a clinical scenario of a presumed SSTI (cellulitis or abscess) and included a unique combination of zero to five interventions (outpatient follow-up, inappropriate antibiotic request flag, thermal imaging for cellulitis or rapid wound MRSA PCR for abscess, patient education/shared decision-making, and clinical decision support). Out of 64 possible vignettes, we asked participants to respond to eight vignettes. Following each vignette, we asked participants if they would prescribe an antibiotic in their everyday practice (yes/no). We built adjusted hierarchical logistic regression models to estimate the probability of prescribing an antibiotic for each intervention and vignette.

**Results:**

Surveys were completed by 113 EM physicians. The thermal imaging, rapid wound MRSA PCR, and patient education/shared decision-making interventions showed the largest decrease (15–20%) in antibiotic prescribing probability. Vignettes with a combination of both a diagnostic intervention (thermal imaging or rapid wound MRSA PCR) and a patient education/shared decision-making intervention had the lowest prescribing probabilities.

**Conclusion:**

We recommend future research focuses on the development and integration of novel diagnostic tools to identify true infection and incorporate shared decision-making to improve diagnosis and management of SSTIs.

## Introduction

Inappropriate antibiotic prescribing often results from diagnostic uncertainty or error. This gap in care quality has been identified as a primary, modifiable contributor to the global increase in antibiotic-resistant bacterial infections.^1^ In the U.S. alone, 2.8 million antibiotic-resistant infections occur each year with 35,000 associated deaths.^2^ Thus, there have been multiple “calls to action” related to antibiotic stewardship, including those highlighting the emergency department (ED).^3–6^

Skin and soft tissue infections (SSTIs) account for approximately two percent of all ED encounters (~3 million annual visits) and available reports indicate frequent suboptimal antibiotic prescribing in this setting.^7–10^ Specifically, cellulitis is overdiagnosed in 30% of cases in which patients with noninfectious mimics, termed pseudocellulitis, are prescribed antibiotics.^8,11,12^ Among cases of accurately diagnosed cellulitis, providers often use two antibiotics despite randomized controlled trials demonstrating this does not improve clinical outcomes.^13–15^ Additionally, uncomplicated abscesses are often treated with one or more antibiotics despite high cure rates being observed with incision and drainage alone across two large RCTs (number needed to treat (NNT) for antibiotics to prevent treatment failure = 14–26).^16–19^

Our previous qualitative work characterized drivers of antibiotic decision-making for SSTIs among emergency medicine (EM) physicians which were then mapped to potential interventions.^20^ Intervention mapping is a systematic framework used for the development, implementation, and evaluation of clinical interventions through incorporation of evidence-based practices and the social and cognitive determinants of clinical decision-making.^21,22^ As there are limited resources to implement stewardship-focused interventions in the ED, we sought to conduct an experiment to quantitatively assess the potential impact of the developed interventions. In order to capture changes in antibiotic prescribing decisions for SSTIs based on these interventions, we utilized case vignettes that presented brief clinical scenarios with various combinations of the proposed interventions and asked EM physicians if they would treat with antibiotics. Case vignettes have historically been used to study clinical decision-making due to their flexibility in manipulating multiple clinical factors while being cost-effective and overcoming ethical limitations of experimental research with real patients.^23^

There is a clear need to evaluate and prioritize interventions that can optimize antibiotic use in the management of skin and soft tissue conditions evaluated in the ED. Thus, the objective of this study was to evaluate the effectiveness of a set of interventions in minimizing inappropriate prescription of antibiotics for presumed SSTIs in the ED using a case vignette survey design to simulate the decision-making process.

## Methods

We conducted a case vignette study via an electronic survey with a national sample of EM physicians. The case vignettes followed a factorial design where the inclusion of each intervention factor was systematically varied, enabling assessment of the relative influence of each intervention on the provider’s decision to prescribe an antibiotic for presumed SSTI.

## Participants

Our target population was practicing EM physicians, which we operationalized as post-residency physicians who self-reported working at least three shifts a month in an ED and who identified EM as their primary specialty. The University of Wisconsin Survey Center facilitated the recruitment of EM physicians for our electronic survey. We purchased a random list of 2000 EM physicians licensed in the U.S. through the American Medical Association from IQVIA. Three percent of surveys were distributed to EM physicians ages 71–80, 13% to EM physicians ages 61–70, 20% to EM physicians ages 51–60, 33% to EM physicians ages 41–50, and 31% to EM physicians ages 31–40. We selected this distribution based on the age distribution of the entire IQVIA dataset and excluding EM physicians > 80 and < 31 to reduce the number of retired or training EM physicians approached. We conducted three rounds of survey outreach from January 2020 to June 2020 and approached a total of 1000 EM physicians with a pre-notification letter including a cash pre-incentive, invitation email, and follow-up emails. The University of Wisconsin Institutional Review Board deemed this study to be exempt.

### Case vignettes

**Figure 1.**
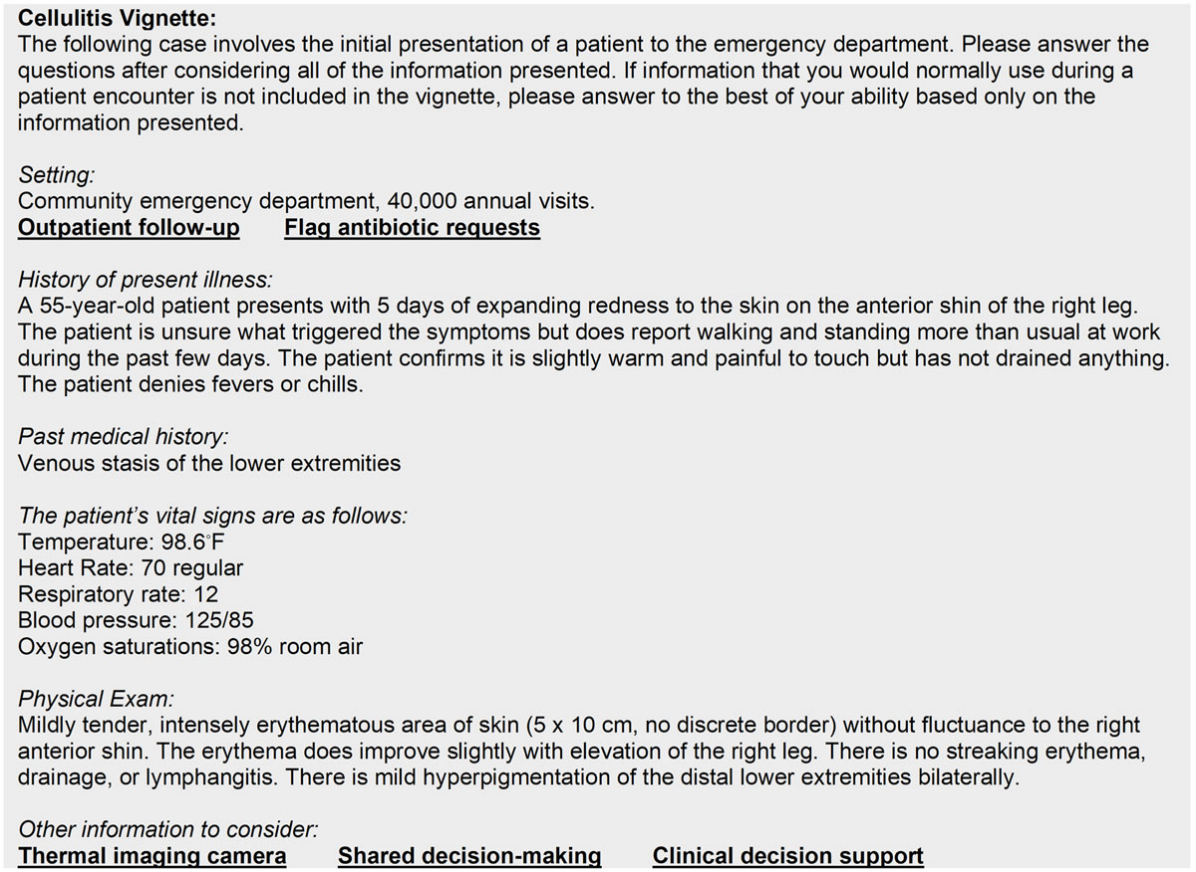
Cellulitis vignette base clinical scenario and mapped interventions.

**Figure 2.**
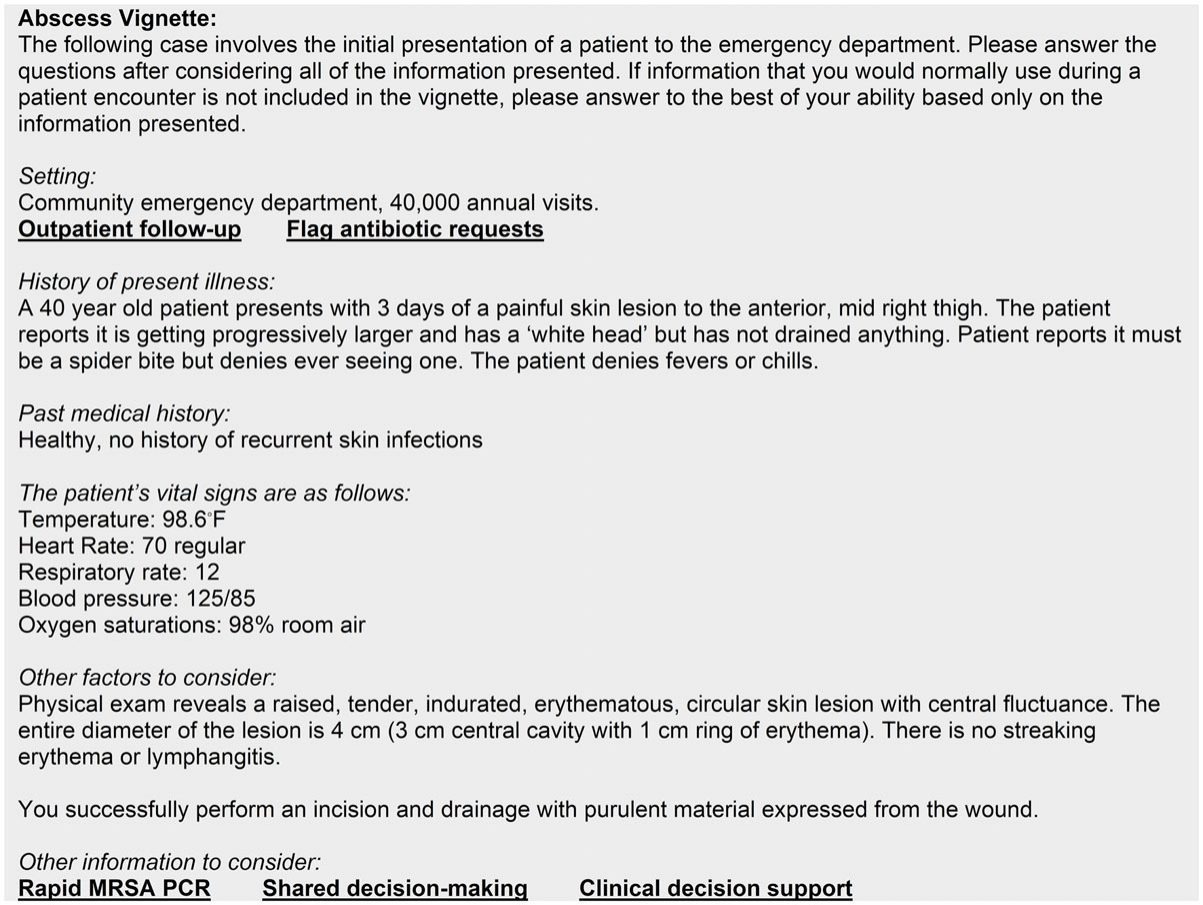
Abscess vignette base clinical scenario and mapped interventions.

The case vignettes consisted of a base clinical scenario for cellulitis (Figure 1) or abscess (Figure 2) which were modifiable by inclusion of the intervention factors. We developed the base clinical scenarios to portray an ambiguous dermatologic complaint, for which the differential would include SSTI, to better capture differences in antibiotic prescribing. We completed pilot testing and cognitive interviewing with EM physicians to get feedback on the content and wording of the case vignettes to ensure they evoked clinical uncertainty.

The case vignettes contained five binary factors that were either present or absent in unique combinations for each vignette. Using a systems-engineering informed qualitative approach, we previously identified barriers to appropriate antibiotic prescribing and mapped interventions to address these barriers.^20^ The five factors included 1) lack of access to care, 2) patient expectations, 3) diagnostic uncertainty, 4) fear of adverse outcomes, and 5) provider knowledge gaps. The phrases in bold and underlined in Figure 1 and Figure 2 indicate where each of the mapped interventions would be inserted into the vignette. Table 1 lists each mapped intervention and its associated factor and clinical scenario by condition.

**Table 1.** Mapped interventions for appropriate antibiotic prescribing for cellulitis and abscess

Mapped Intervention	Condition	Factor	Intervention Wording in Vignette
Community paramedicine program or telehealth for outpatient follow-up	Cellulitis	Lack of Access to Care	Your emergency department can arrange 24-hour follow-up for discharged patients by either an in-home community paramedicine (paramedic) visit or a virtual appointment using an online, video enabled telehealth system.
	Abscess		
Create provider flag for encounters involving inappropriate antibiotic prescription requests	Cellulitis	Patient Expectations	Your hospital quality department allows you to flag cases involving inappropriate requests for antibiotics and these are excluded from your patient satisfaction metrics.
	Abscess		
Thermal imaging camera	Cellulitis	Diagnostic Uncertainty	A thermal imaging camera indicates the maximum skin surface temperature of the affected leg is identical to the unaffected leg. The average reported skin temperature difference for cellulitis is 3.7^°^C greater in the affected limb.^28^
MRSA PCR of wound with rapid turnaround	Abscess		You obtain a rapid PCR swab of the purulent drainage expressed during the I+D procedure which resulted negative for MRSA (PCR negative predictive value = 95%).
Patient educational materials or shared decision tool which outlines risks/benefits of antibiotics for specific clinical scenario	Cellulitis	Fear of Adverse Outcomes	The patient asks you if there is any way to manage this without an antibiotic due to concerns over side effects and *Clostridiodes difficile*.
	Abscess		
Clinical decision support/BPA	Cellulitis	Provider Knowledge Gaps	A best practice alert in your electronic health record has triggered the following message, “Studies indicate up to 30% of cellulitis cases diagnosed in the emergency department are actually misdiagnosed mimics which do not require antibiotics.”^8,36^
	Abscess		Your electronic health record has alerted you that this condition can be safely managed without antibiotics in the majority of cases (Number needed to treat (NNT) with antibiotics to prevent 1 treatment failure = 14-26).^18,19^

All combinations of the five interventions being present/absent produced 32 case vignettes per condition, with 64 case vignettes in total. Which factors are included in each numbered vignette is described in a design matrix (Supplement Table 1). It was unrealistic to ask participants to read 64 vignettes, so we utilized a balanced incomplete block design where participants responded to 8 vignettes, 4 for cellulitis and 4 for abscess.^24^ We chose 4 vignettes per condition to balance participant burden (response rate) and factor coverage. The respondents for each factor block (e.g. *10001* factor block: first and fifth factors are present) were shown both cellulitis and abscess vignettes for that assigned block.

We randomized which four factor blocks were presented to participants and whether a cellulitis or abscess vignette was presented first. The survey then alternated between presenting a cellulitis or abscess vignette. We randomized the order within the lineup of cellulitis and abscess vignettes such that the cellulitis and abscess vignettes of the same factor block may or may not have ended up next to each other in the survey (Supplement Figure 1).

Following each vignette, we asked participants to indicate how likely they were to prescribe antibiotics for that case (not very likely, slightly likely, somewhat likely, very likely, extremely likely) and if they would prescribe an antibiotic in their everyday practice (yes/no). For cellulitis cases only, we also asked participants how likely it was that the patient had cellulitis (not very likely, slightly likely, somewhat likely, very likely, extremely likely).

### Statistical analysis

We described the cohort of survey respondents according to their basic demographics, including gender (female, male, not reported), ethnicity (Hispanic/Latino, not Hispanic/Latino, not reported), race (American Indian or Alaskan Native, Asian or Hmong, Black or African American, Native Hawaiian or Other Pacific Islander, White, other), training (American Board of Emergency Medicine Certified (yes/no), years of post-residency clinical experience (<5, 5 to 9, 10 to 14, 15 to 19, 20 to 24, 25+), and characteristics of their primary practice site (ED setting (urban, suburban, rural), type of ED (community, academic, veterans affairs, critical access, other), U.S. census region (Midwest, Northeast, South, West).

Utilizing judgment analysis methods, we built multilevel logistic regression models for each participant to infer how they weighed the intervention factors in their decision whether to prescribe an antibiotic. The analysis consisted of a hierarchical logistic model possessing two regression equations, one modeling the vignette effects within the participants, and the other modeling participant effects between participants (see Supplement Material 1 for model specification).^25–27^

We conducted two analyses according to these methods in order to estimate antibiotic prescribing probabilities, one to estimate probabilities for each factor and one to estimate probabilities for each vignette. The factor analysis incorporated indicator variables for each of the five factors into the model so that individual vignettes could be represented by the presence/absence of each factor. Alternatively, the vignette analysis included a variable for the vignette number (1–32). Both analyses included a cellulitis/abscess indicator and interaction terms between the cellulitis/abscess indicator and factor indicators or vignette number so prescribing probabilities for each condition could be distinguished. The factor model aggregated the prescribing probabilities across vignettes with each factor to estimate the overall prescribing probability per factor. The vignette model simply produced individual prescribing probabilities for each of the 64 case vignettes (32 per infection type). We adjusted for the following covariates in both models: years of experience, confidence in diagnosis, attitude about inappropriate antibiotic prescribing, self-reported inappropriate antibiotic prescribing behavior, and familiarity with the Infectious Diseases Society of America antibiotic prescribing guidelines.

In a final analysis of the cumulative effect of including multiple mapped interventions, irrespective of the specific interventions, we categorized the vignettes as having zero, one, two, three, four, or five intervention factors present. For instance, the cellulitis vignette with the thermal imaging camera intervention and clinical decision support intervention would be considered a vignette with two intervention factors. We then calculated the proportion of participants that would prescribe an antibiotic for vignettes grouped by number of factors and condition and assessed differences in prescribing via a Chi-square test. All analyses were conducted in Stata 17.

## Results

Of the 1000 survey invitations sent out, 139 surveys were started (13.9% response rate) and 113 surveys were completed (13 respondents were ineligible, 13 surveys were incomplete). Participants were primarily male (71.6%), not Hispanic/Latino (90.3%), White (84.1%), and American Board of Emergency Medicine certified (85.8%) with 20 or more years post-residency clinical experience (51.4%) (Table 2). Participants most commonly worked at suburban (40.7%) and community EDs (69.0%) in the Midwest (38.1%).

**Table 2.** Physician and practice setting characteristics (n = 113)

Self-reported Gender	
Female	30 (26.6%)
Male	81 (71.6%)
Not Reported	2 (1.8%)
Ethnicity	
Hispanic/Latino	7 (6.2%)
Not Hispanic/Latino	102 (90.3%)
Not Reported	4 (3.5%)
Race	
American Indian or Alaskan Native	1 (0.9%)
Asian or Hmong	8 (7.1%)
Black or African American	3 (2.7%)
Native Hawaiian or Other Pacific Islander	1 (0.9%)
White	95 (84.1%)
Other	3 (2.7%)
American Board of Emergency Medicine Certified	
Yes	97 (85.8%)
# Years Post Residency	
Less than 5	4 (3.5%)
5 to 9	19 (16.8%)
10 to 14	14 (12.4%)
15 to 19	18 (15.9%)
20 to 24	29 (25.7%)
25 or more	29 (25.7%)
Setting	
Rural	22 (19.5%)
Suburban	46 (40.7%)
Urban	45 (39.8%)
Type of Emergency Department	
Academic	30 (26.6%)
Community	78 (69.0%)
Critical Access	1 (0.9%)
Veterans Affairs	2 (1.8%)
Other	2 (1.8%)
U.S. Census Region	
Midwest	43 (38.1%)
Northeast	20 (17.7%)
South	23 (20.4%)
West	27 (23.9%)

Each of the 32 vignettes had a median of 14 (Min: 11, Max: 16) responses (Supplement Table 1) with each intervention appearing 224 to 228 times per condition. Across all vignettes, participants would have been prescribed antibiotics in 39.2% of cellulitis cases and 59.3% of abscess cases.

A response of “A great deal” to “How much of a problem is inappropriate antibiotics in the emergency department?” was associated with significantly lower antibiotic prescribing than participants responding “A little” with an odds ratio (OR) of 0.127 (95% CI: 0.028 – 0.588, p = 0.01) in the factor model with similar results in the vignette model. More years of clinical experience was also associated with lower antibiotic prescribing in the factor model (and similarly in the vignette model) with 20 to 24 years (OR 0.134 95% CI: 0.023 – 0.797, p = 0.03) and 25 or more years (OR 0.137 95% CI: 0.024 – 0.782, p = 0.03) of clinical experience having significantly lower prescribing than participants with less than five years of experience (Supplement Table 2 and 3).

**Figure 3.**
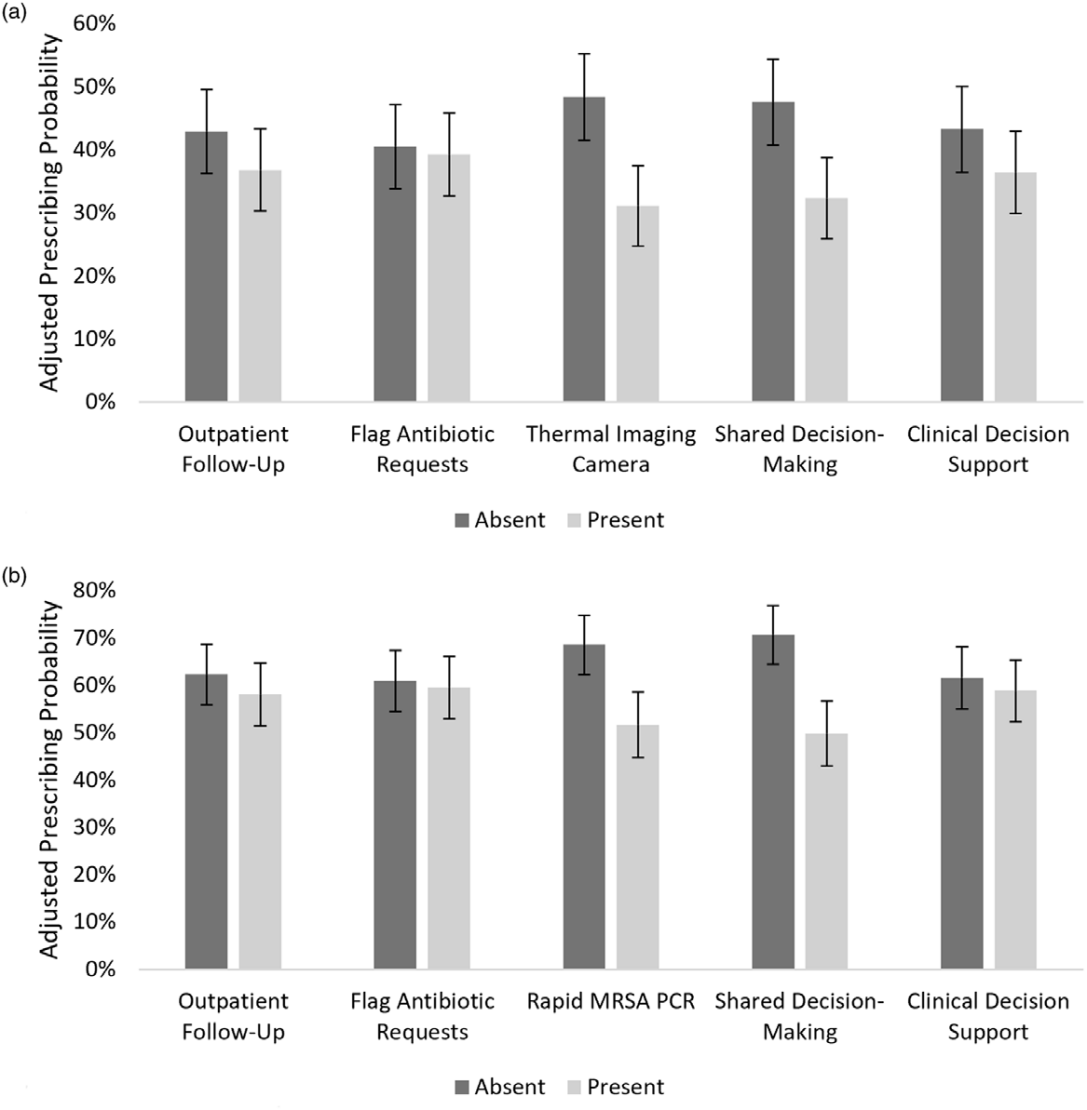
Adjusted prescribing probabilities by mapped interventions for cellulitis (Figure 3a) and abscess (Figure 3b).

Figure 3a compares the adjusted antibiotic prescribing probabilities for cellulitis when the five mapped interventions were present versus absent in the case vignettes. The thermal imaging camera intervention showed the largest decrease in antibiotic prescribing probability of 17.2% (95% CI: 16.7% – 17.7%). The patients raising concern over potential antibiotic side effects/adverse reactions (representing education materials or a shared decision-making intervention) had a similarly large decrease in antibiotic prescribing probability of 15.2% (95% CI: 14.8% – 15.6%). The remaining interventions of paramedicine or telehealth follow-up, inappropriate antibiotic prescribing flag, and clinical decision support had smaller decreases in antibiotic prescribing probability of 6.1% (95% CI: 5.9% – 6.3%), 1.3% (95% CI: 1.2% – 1.4%), and 6.8% (95%CI: 6.6% – 7.1%) respectively.

Figure 3b compares the adjusted antibiotic prescribing probabilities for abscess when the five mapped interventions were present versus absent in the case vignettes. The largest decrease in antibiotic prescribing probability for abscess was 20.9% (95% CI: 20.2% – 21.5%) for the patient education materials or shared decision-making intervention followed by 16.9% (95% CI: 16.3% – 17.5%) for the rapid MRSA PCR intervention. As with cellulitis, the interventions of paramedicine or telehealth follow-up, inappropriate antibiotic prescribing flag, and clinical decision support had smaller decreases in antibiotic prescribing probability for abscess of 4.2% (95% CI: 4.0% – 4.4%), 1.3% (95% CI: 1.2% – 1.4%), and 2.7% (95%CI: 2.7% – 2.8%) respectively.

**Figure 4.**
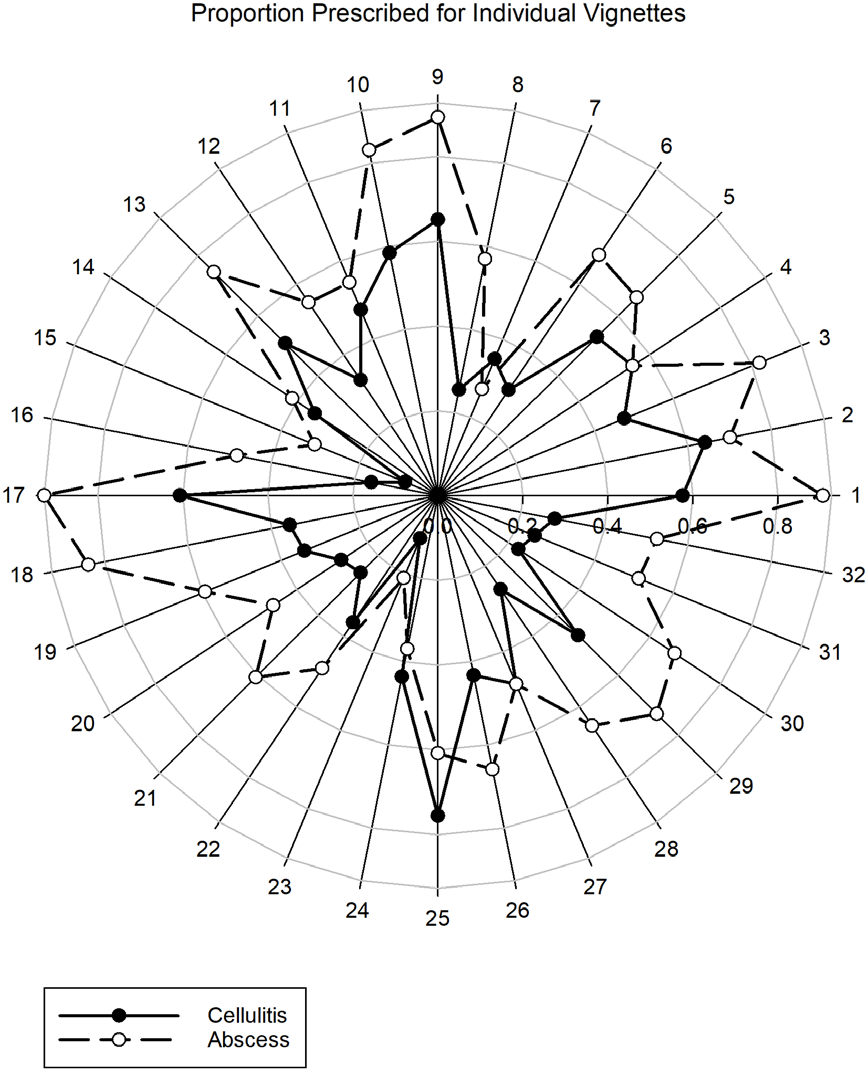
Radar plot of adjusted prescribing probabilities by vignette for cellulitis and abscess.

Figure 4 displays the adjusted antibiotic prescribing probabilities for all 64 case vignettes in a radar plot. The numbers 1 to 32 circling the plot indicate the vignette number (see associated interventions for each vignette number in Supplement Table 1) with the adjusted antibiotic prescribing probabilities plotted radially with 0% probability at the center and extending to 100% probability at the edge. This shows that in the majority of cases, participants had a higher probability of prescribing antibiotics for abscesses compared to cellulitis. The figure also demonstrates that the modification of the case vignettes through the inclusion of the mapped interventions resulted in considerable differences in antibiotic prescribing behavior.

The abscess case vignettes with the four lowest adjusted antibiotic prescribing probabilities (23, 7, 15, 24) all included both the patient education materials or shared decision-making intervention and the rapid MRSA PCR intervention. Similarly, the cellulitis case vignettes with the three lowest adjusted antibiotic prescribing probabilities (15, 23, 16) all included both the patient education materials or shared decision-making intervention and the thermal imaging camera intervention.

**Figure 5.**
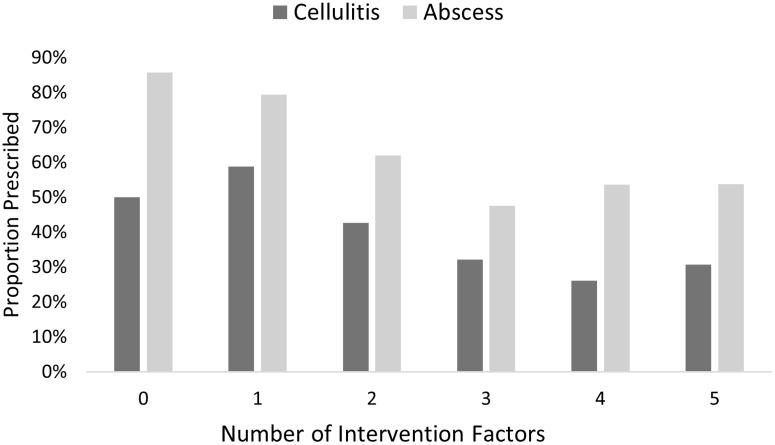
Proportion prescribed antibiotics for cellulitis and abscess by number of intervention factors.

The proportion of participants that would prescribe antibiotics for vignettes with zero, one, two, three, four, or five intervention factors present was significantly different for both cellulitis (χ^2^ = 20.8, *P* = 0.001) and abscess (χ^2^ = 25.2, *P* < 0.001) (Figure 5). Note that the number of responses for each category of zero to five intervention factors present was quite different due to the number of ways vignettes could be constructed (e.g. one vignette with zero intervention factors vs. ten vignettes with three intervention factors). The lowest proportion of antibiotics were prescribed for vignettes with four intervention factors for cellulitis (26.1%) and for vignettes with three intervention factors for abscess (47.6%).

## Discussion

To our knowledge, this study represents the first judgment analysis applied to hierarchical prioritization of antibiotic stewardship interventions. Our vignette design effectively facilitated judgment analysis as demonstrated by the variety of prescribing responses elicited by the different vignettes. The significant difference in proportion prescribed by number of factors also shows that manipulation of the factors produced different prescribing decisions. Our overall findings indicate that the application of diagnostic tools for skin and soft tissue infections has the highest potential for reducing inappropriate antibiotic prescribing. This finding reinforces the documented difficulty in distinguishing cellulitis from pseudocellulitis and the need for novel diagnostic methods to assist in differential diagnosis.^12,28^

Skin surface thermal imaging allows for potential real-time testing that can decrease misdiagnosis and prevent unnecessary antibiotic prescribing. Recently, our team published a large diagnostic validation study which found that differences in detection of skin surface temperature through thermal imaging, with or without the additional predictive tool ALT-70 (asymmetry, leukocytosis, tachycardia, and age ≥70 years) would result in improved diagnosis of cellulitis and could serve as a valuable tool to decrease overdiagnosis in the ED.^12^ Also in agreement with our findings, prior research has found rapid MRSA PCR testing following incision and drainage of an abscess improves appropriate usage and de-escalation of anti-MRSA antibiotic agents.^29,30^

The other highly effective intervention in reducing antibiotic prescribing for cellulitis and abscess was patients raising concerns for potential side effects/adverse reactions (e.g. *Clostridiodes difficile*). This intervention was designed to represent the provision of educational materials and/or shared decision-making tools that encourage patients to directly ask the provider about the potential risks and benefits of antibiotics. Increasingly the importance of patient engagement in the diagnostic process has been recognized as essential to improving patient care.^31–34^ Qualitative data shows that patients want to be involved in the decisions surrounding their care in the ED, but often do not feel empowered to do so.^33^ Additionally, there are EM physician-identified barriers that make it challenging to consistently engage in shared decision-making with patients.^35^ There is a paucity of literature examining how to pragmatically implement patient engagement strategies like shared decision-making in the ED for infectious conditions and their downstream impact on diagnostic and antimicrobial stewardship metrics.

The community paramedicine program or telehealth follow-up, provider flag for inappropriate antibiotic prescription requests, and clinical decision support interventions had a smaller impact on EM physician antibiotic prescribing decisions than diagnostic tools or patient education. We propose that these interventions have potential and should be considered as part of intervention bundles but would be lower on the prioritization list as individual interventions.

It is important to highlight the limitations of our study. First, physicians made decisions based on case vignettes that do not contain all clinically relevant factors that would be present in a real case. Additionally, the influence of interacting with a patient face-to-face could not be replicated in the vignettes. Another limitation was that the second and third rounds of survey distribution occurred during the first four months of the COVID-19 pandemic in the U.S. Response rates may have been lower due to the burden placed on EM physicians by the pandemic. The observed low participation from providers working in critical access settings is likely due to our inclusion criteria requiring primary specialization in emergency medicine. It is important to note that these findings may not be generalizable to settings with limited resources or primarily staffed by providers without specialization in emergency medicine. Finally, our sample size was too small to incorporate the interactions between factors in our models to directly assess the impact of combinations of interventions. However, we found the case vignettes with the lowest adjusted prescribing probabilities for both abscess and cellulitis included the patient education materials or shared decision-making intervention and diagnostic tool (rapid MRSA PCR or thermal imaging camera) intervention, indicating the combination of these two interventions may be the most effective in reducing inappropriate antibiotic prescribing.

Our study successfully identified several rigorously designed ED antibiotic stewardship interventions for SSTIs that had a significant impact on prescribing behavior in simulated case vignettes. Prioritization of stewardship interventions in experimental contexts prior to implementation is critical to preserve resources and optimize the likelihood of real-world effectiveness. Based on our results, we recommend future research focused on the development and integration of novel diagnostic tools, such as thermal imaging, and interventions focused on patient engagement and shared decision-making to improve diagnosis and management of SSTIs in the ED.

## Supporting information

Griffin et al. supplementary material Griffin et al. supplementary material

## Supplementary material

The supplementary material for this article can be found at https://doi.org/10.1017/ice.2024.211
