# Supplementary material for: Prioritizing emergency department antibiotic stewardship interventions for skin and soft tissue infections using judgment analysis
Source: Infect Control Hosp Epidemiol. 2025 Jan 20;46(3):272–80. doi: 10.1017/ice.2024.211 (PMC11883652; doi:10.1017/ice.2024.211)
Supplement: Griffin et al. supplementary material [file S0899823X24002113sup001.pdf]

Supplementary Material

**Supplement Figure 1:** Balanced incomplete block design example	p. 2
**Supplement Material 1:** Specification of the model	pp. 3-4
**Supplement Table 1:** 32 x 5 design matrix	p. 5
**Supplement Table 2:** Hierarchical logistic model vignette logits by condition	pp. 6-8
**Supplement Table 3:** Hierarchical logistic model factor logits by condition	pp. 9-10

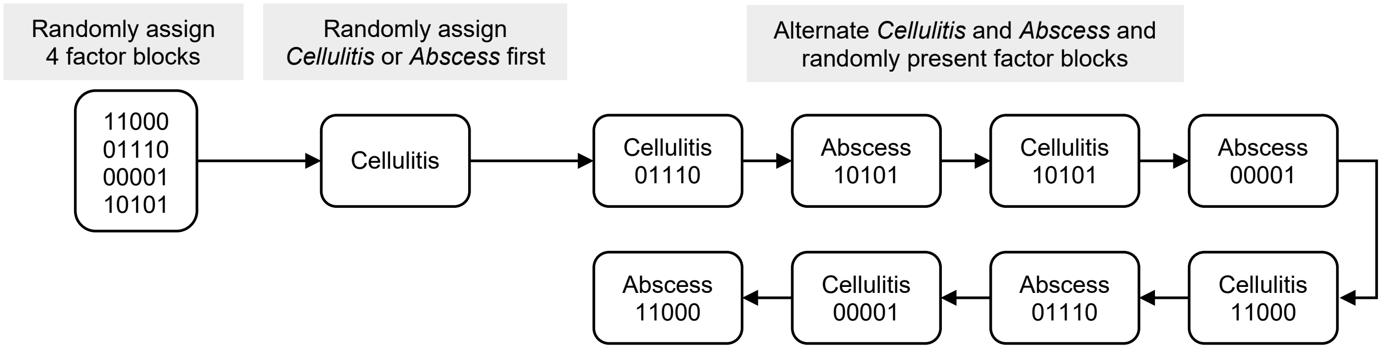

Supplement Figure 1. Balanced incomplete block design example

Supplementary Material 1. Specification of the model

First, for each participant we modeled a separate within-participant regression model:

$$\operatorname{logit}\left(\pi_{ij}\right) = \beta_{i0} + \sum_{p=1}^{P} \beta_{ip} x_{ijp} \qquad (1)$$

where

p_{ij} = the prescribing probability for vignette i by physician j,

x_{ijp} = the value of the vignette characteristics for vignette i and participant j,

b_{ip} = the regression coefficients within participant j,

for

i = 1, 2, ..., k vignettes,

j = 1, 2, ..., n participants,

p = 0, 1, ..., p vignette variables.

Physician responses on the participant level are subsequently predicted by the values of the corresponding vignette characteristics. Second, each of the regression coefficients from the above model may be represented as a between-participants model:

$$\beta_{im} = \gamma_{0m} + \gamma_{1m} z_{1i} + \cdots + \gamma_{rm} z_{ri} + u_{im} \qquad (2)$$

where

b_{im} = the within-participants regression coefficient for vignette characteristic m and physician participant i,

z_{ri} = the values of the participant characteristics for physician participant i,

g_{rm} = the regression coefficient that describes the effects of participant variables on the within-participants relationships b_{im},

u_{im} = random errors.

Equations (1) and (2) may be written as a single equation:

$$y_{ij} = \text{logit}\left(\pi_{ij}\right) = \left[\sum_{r=0}^{q}\sum_{m=0}^{p}\gamma_{rm} z_{ri}\right] + \left[\sum_{m=0}^{p} u_{im} + \varepsilon_{ij}\right] \qquad (3)$$

The observed (0,1) prescribing response, at level 1, is y_{ij} ~ *Bin*(1, p_{ij}) with Binomial variance p_{ij} (1-p_{ij}). This assumption of binomial variation can be tested by fitting an ‘extra-binomial parameter’ s^2_e , so the vignette-level variance would be s^2_e p_{ij} (1-p_{ij}). Estimates close to 1.00 indicate appropriateness of the binomial assumption.

Supplement Table 1. 32 x 5 design matrix

Vignette #	Number of Responses	Lack of access to care	Patient expectations	Diagnostic uncertainty	Fear of adverse outcomes	Provider knowledge gaps
1	14	0	0	0	0	0
2	14	0	0	0	0	1
3	14	0	0	0	1	0
4	15	0	0	0	1	1
5	15	0	0	1	0	0
6	12	0	0	1	0	1
7	13	0	0	1	1	0
8	14	0	0	1	1	1
9	14	0	1	0	0	0
10	16	0	1	0	0	1
11	15	0	1	0	1	0
12	13	0	1	0	1	1
13	15	0	1	1	0	0
14	14	0	1	1	0	1
15	14	0	1	1	1	0
16	15	0	1	1	1	1
17	11	1	0	0	0	0
18	15	1	0	0	0	1
19	16	1	0	0	1	0
20	14	1	0	0	1	1
21	14	1	0	1	0	0
22	15	1	0	1	0	1
23	14	1	0	1	1	0
24	14	1	0	1	1	1
25	14	1	1	0	0	0
26	15	1	1	0	0	1
27	16	1	1	0	1	0
28	12	1	1	0	1	1
29	14	1	1	1	0	0
30	13	1	1	1	0	1
31	15	1	1	1	1	0
32	13	1	1	1	1	1

0 = Factor absent, 1 = Factor present

Supplement Table 2. Hierarchical logistic model vignette logits by condition

		Coefficient	Std. err.	z	P>|z|	95% Conf. Interval	
Condition							
	Cellulitis (Reference)	Ref	Ref	Ref	Ref	Ref	Ref
	Abscess	2.66	1.15	2.32	0.02	0.41	4.91
Vignette							
	Vignette 1	Ref	Ref	Ref	Ref	Ref	Ref
	Vignette 2	0.39	0.96	0.41	0.68	-1.48	2.27
	Vignette 3	-0.59	0.97	-0.61	0.54	-2.49	1.31
	Vignette 4	-0.15	0.96	-0.15	0.88	-2.03	1.74
	Vignette 5	-0.27	0.97	-0.28	0.78	-2.18	1.63
	Vignette 6	-1.67	1.07	-1.56	0.12	-3.76	0.43
	Vignette 7	-1.35	1.03	-1.30	0.19	-3.37	0.68
	Vignette 8	-1.99	1.04	-1.91	0.06	-4.03	0.05
	Vignette 9	0.46	1.02	0.44	0.66	-1.55	2.46
	Vignette 10	0.05	0.97	0.05	0.96	-1.86	1.96
	Vignette 11	-0.59	0.98	-0.60	0.55	-2.52	1.33
	Vignette 12	-1.48	1.01	-1.46	0.14	-3.47	0.51
	Vignette 13	-0.40	0.95	-0.42	0.68	-2.26	1.47
	Vignette 14	-1.35	0.99	-1.37	0.17	-3.28	0.58
	Vignette 15	-3.74	1.41	-2.66	0.01	-6.50	-0.98
	Vignette 16	-2.79	1.11	-2.51	0.01	-4.97	-0.61
	Vignette 17	0.19	1.02	0.18	0.86	-1.81	2.18
	Vignette 18	-1.31	1.01	-1.30	0.19	-3.28	0.66
	Vignette 19	-1.40	0.97	-1.45	0.15	-3.30	0.49
	Vignette 20	-1.85	1.02	-1.81	0.07	-3.85	0.15
	Vignette 21	-1.97	1.03	-1.91	0.06	-3.99	0.05
	Vignette 22	-1.28	0.98	-1.31	0.19	-3.20	0.64
	Vignette 23	-3.36	1.43	-2.35	0.02	-6.16	-0.56
	Vignette 24	-0.82	0.98	-0.84	0.40	-2.74	1.10
	Vignette 25	1.17	1.05	1.10	0.27	-0.90	3.23
	Vignette 26	-0.84	0.94	-0.89	0.37	-2.69	1.01
	Vignette 27	-0.55	0.99	-0.56	0.58	-2.50	1.39
	Vignette 28	-1.90	1.11	-1.72	0.09	-4.07	0.26
	Vignette 29	-0.64	1.03	-0.62	0.53	-2.65	1.37
	Vignette 30	-2.19	1.09	-2.01	0.04	-4.33	-0.06
	Vignette 31	-2.05	1.04	-1.97	0.05	-4.08	-0.01
	Vignette 32	-1.80	1.08	-1.68	0.09	-3.91	0.31
Vignette X Condition							
	Vignette 1 X Abscess	Ref	Ref	Ref	Ref	Ref	Ref
	Vignette 2 X Abscess	-2.27	1.45	-1.57	0.12	-5.11	0.56
	Vignette 3 X Abscess	-0.38	1.55	-0.25	0.80	-3.42	2.65
	Vignette 4 X Abscess	-2.66	1.44	-1.85	0.07	-5.49	0.16
	Vignette 5 X Abscess	-1.87	1.46	-1.28	0.20	-4.72	0.99
	Vignette 6 X Abscess	-0.33	1.54	-0.22	0.83	-3.35	2.69
	Vignette 7 X Abscess	-3.18	1.54	-2.07	0.04	-6.19	-0.16

Vignette 8 X Abscess	-0.72	1.48	-0.48	0.63	-3.61	2.18
Vignette 9 X Abscess	-0.64	1.74	-0.37	0.71	-4.04	2.76
Vignette 10 X Abscess	-0.91	1.50	-0.61	0.55	-3.86	2.04
Vignette 11 X Abscess	-2.25	1.46	-1.55	0.12	-5.11	0.60
Vignette 12 X Abscess	-1.34	1.49	-0.90	0.37	-4.26	1.57
Vignette 13 X Abscess	-1.17	1.45	-0.81	0.42	-4.01	1.67
Vignette 14 X Abscess	-2.27	1.45	-1.57	0.12	-5.11	0.57
Vignette 15 X Abscess	-0.49	1.78	-0.28	0.78	-3.97	2.99
Vignette 16 X Abscess	-0.42	1.53	-0.27	0.78	-3.42	2.58
Vignette 17 X Abscess	0.14	1.79	0.08	0.94	-3.36	3.64
Vignette 18 X Abscess	0.51	1.52	0.34	0.74	-2.47	3.49
Vignette 19 X Abscess	-1.16	1.45	-0.80	0.43	-4.00	1.69
Vignette 20 X Abscess	-1.45	1.47	-0.99	0.32	-4.33	1.43
Vignette 21 X Abscess	-0.51	1.50	-0.34	0.73	-3.46	2.43
Vignette 22 X Abscess	-1.88	1.45	-1.30	0.19	-4.72	0.96
Vignette 23 X Abscess	-1.63	1.88	-0.87	0.39	-5.31	2.05
Vignette 24 X Abscess	-3.06	1.46	-2.10	0.04	-5.92	-0.20
Vignette 25 X Abscess	-3.63	1.53	-2.37	0.02	-6.64	-0.63
Vignette 26 X Abscess	-1.32	1.42	-0.93	0.35	-4.10	1.47
Vignette 27 X Abscess	-2.66	1.47	-1.81	0.07	-5.55	0.22
Vignette 28 X Abscess	-0.29	1.55	-0.19	0.85	-3.33	2.75
Vignette 29 X Abscess	-1.05	1.56	-0.67	0.50	-4.12	2.01
Vignette 30 X Abscess	0.11	1.55	0.07	0.95	-2.94	3.15
Vignette 31 X Abscess	-0.99	1.48	-0.67	0.50	-3.90	1.91
Vignette 32 X Abscess	-1.15	1.54	-0.75	0.45	-4.16	1.86
Years Clinical Experience						
Less than 5	Ref	Ref	Ref	Ref	Ref	Ref
5 to 9	-1.65	1.02	-1.62	0.11	-3.65	0.35
10 to 14	-1.54	1.01	-1.52	0.13	-3.53	0.45
15 to 19	-1.69	1.06	-1.60	0.11	-3.76	0.39
20 to 24	-2.15	0.99	-2.17	0.03	-4.10	-0.21
25 or more	-2.15	0.97	-2.22	0.03	-4.05	-0.25
Confidence in cellulitis diagnosis						
Not at all confident	Ref	Ref	Ref	Ref	Ref	Ref
Slightly confident	-0.91	1.45	-0.63	0.53	-3.75	1.93
Somewhat confident	-0.53	1.40	-0.38	0.71	-3.27	2.21
Very confident	-0.06	1.54	-0.04	0.97	-3.08	2.97
Confidence in determining if abscess would benefit from antibiotics						
Not at all confident	Ref	Ref	Ref	Ref	Ref	Ref
Slightly confident	0.83	1.63	0.51	0.61	-2.36	4.02
Somewhat confident	0.24	1.59	0.15	0.88	-2.87	3.35
Very confident	0.45	1.63	0.27	0.78	-2.75	3.65
Extremely confident	0.06	1.76	0.04	0.97	-3.39	3.52
How much of a problem is inappropriate antibiotics in the emergency department						

A little	Ref	Ref	Ref	Ref	Ref	Ref
Somewhat	-1.34	0.82	-1.63	0.10	-2.95	0.27
Quite a bit	-1.58	0.83	-1.90	0.06	-3.21	0.05
A great deal	-2.23	0.86	-2.61	0.01	-3.91	-0.56
How often prescribe antibiotics when not clearly indicated						
Never	Ref	Ref	Ref	Ref	Ref	Ref
Rarely	-0.22	0.75	-0.29	0.77	-1.69	1.25
Sometimes	0.28	0.78	0.36	0.72	-1.25	1.80
Very often	1.17	1.29	0.90	0.37	-1.36	3.70
How familiar with IDSA guidelines for antibiotic prescribing for SSTIs						
Not at all	Ref	Ref	Ref	Ref	Ref	Ref
A little	-0.29	0.65	-0.44	0.66	-1.56	0.98
Somewhat	-0.25	0.62	-0.40	0.69	-1.47	0.97
Very	-1.25	0.66	-1.89	0.06	-2.55	0.05
Extremely	-1.15	1.80	-0.64	0.52	-4.69	2.38
Intercept	4.34	1.89	2.30	0.02	0.63	8.04
Participant Level Variance	1.91	0.49			1.16	3.16
McKelvey & Zavoina Pseudo-R2 (fixed & random effects)	0.53					

IDSA: Infectious Disease Society of America; SSTI: Skin and soft tissue infection

Supplement Table 3. Hierarchical logistic model factor logits by condition

		Coefficient	Std. err.	z	P>|z|	95% Conf. Interval	
Condition							
	Cellulitis (Reference)	Ref	Ref	Ref	Ref	Ref	Ref
	Abscess	1.22	0.42	2.93	0.00	0.40	2.03
Factor							
	Factor 1	-0.38	0.25	-1.53	0.13	-0.86	0.11
	Factor 2	-0.08	0.24	-0.33	0.74	-0.56	0.40
	Factor 3	-1.04	0.25	-4.19	0.00	-1.53	-0.56
	Factor 4	-0.93	0.25	-3.72	0.00	-1.42	-0.44
	Factor 5	-0.42	0.25	-1.70	0.09	-0.90	0.06
Factor X Condition							
	Factor 1 X Abscess	0.12	0.33	0.35	0.72	-0.53	0.76
	Factor 2 X Abscess	0.00	0.33	-0.01	1.00	-0.65	0.64
	Factor 3 X Abscess	0.01	0.33	0.04	0.97	-0.64	0.66
	Factor 4 X Abscess	-0.34	0.33	-1.03	0.30	-0.99	0.31
	Factor 5 X Abscess	0.25	0.33	0.76	0.45	-0.40	0.90
Years Clinical Experience							
	Less than 5	Ref	Ref	Ref	Ref	Ref	Ref
	5 to 9	-1.61	0.94	-1.71	0.09	-3.45	0.24
	10 to 14	-1.37	0.94	-1.47	0.14	-3.21	0.46
	15 to 19	-1.38	0.97	-1.42	0.16	-3.29	0.53
	20 to 24	-2.01	0.91	-2.21	0.03	-3.80	-0.23
	25 or more	-1.99	0.89	-2.23	0.03	-3.74	-0.24
Confidence in cellulitis diagnosis							
	Not at all confident	Ref	Ref	Ref	Ref	Ref	Ref
	Slightly confident	-1.04	1.33	-0.78	0.44	-3.64	1.57
	Somewhat confident	-0.62	1.29	-0.48	0.63	-3.14	1.90
	Very confident	-0.14	1.42	-0.10	0.92	-2.92	2.64
Confidence in determining if abscess would benefit from antibiotics							
	Not at all confident	Ref	Ref	Ref	Ref	Ref	Ref
	Slightly confident	0.66	1.50	0.44	0.66	-2.28	3.59

Somewhat confident	0.14	1.46	0.10	0.92	-2.72	3.01
Very confident	0.28	1.50	0.19	0.85	-2.66	3.23
Extremely confident	-0.23	1.62	-0.14	0.89	-3.41	2.95
How much of a problem is inappropriate antibiotics in the emergency department						
A little	Ref	Ref	Ref	Ref	Ref	Ref
Somewhat	-1.26	0.76	-1.67	0.10	-2.75	0.22
Quite a bit	-1.39	0.76	-1.82	0.07	-2.89	0.11
A great deal	-2.06	0.78	-2.63	0.01	-3.60	-0.53
How often prescribe antibiotics when not clearly indicated						
Never	Ref	Ref	Ref	Ref	Ref	Ref
Rarely	-0.31	0.69	-0.44	0.66	-1.66	1.05
Sometimes	0.17	0.72	0.23	0.82	-1.24	1.58
Very often	1.05	1.19	0.88	0.38	-1.28	3.37
How familiar with IDSA guidelines for antibiotic prescribing for SSTIs						
Not at all	Ref	Ref	Ref	Ref	Ref	Ref
A little	-0.20	0.60	-0.34	0.74	-1.37	0.97
Somewhat	-0.27	0.57	-0.47	0.64	-1.39	0.85
Very	-1.10	0.61	-1.80	0.07	-2.30	0.10
Extremely	-1.15	1.68	-0.68	0.49	-4.44	2.14
Intercept	4.68	1.64	2.86	0.00	1.48	7.89
Participant Level Variance	1.62	0.41			0.99	2.65
McKelvey & Zavoina Pseudo-R2 (fixed & random effects)	0.44					

IDSA: Infectious Disease Society of America; SSTI: Skin and soft tissue infection
